# Comparison of Characteristics of Human Amniotic Membrane and Human Adipose Tissue Derived Mesenchymal Stem Cells

**Published:** 2017-01

**Authors:** Khadijeh Dizaji Asl, Hajar Shafaei, Jafar Soleimani Rad, Hojjat Ollah Nozad

**Affiliations:** Department of Anatomical Science, Tabriz University of Medical Sciences, Tabriz, Iran

**Keywords:** Adipose tissue, Amniotic membrane, CD 271, Mesenchymal stem cells

## Abstract

**BACKGROUND:**

Mesenchymal stem cells (MSCs) are ideal candidates for treatment of diseases. Amniotic membranes are an inexpensive source of MSCs (AM-MSC) without any donor site morbidity in cell therapy. Adipose tissue derived stem cells (ASCs) are also suitable cells for cell therapy. There is discrepancy in CD271 expression among MSCs from different sources. In this study, the characteristics of AM-MSC and ASCs and CD271 expression were compared.

**METHODS:**

Adult adipose tissue samples were obtained from patients undergoing elective surgical procedure, and samples of amniotic membrane were collected immediately after caesarean operation. After isolation and expansion of MSCs, the proliferation rate and viability of cells were evaluated through calculating DT and MTT assay. Expression of routine mesenchymal specific surface antigens of MSCs and CD271 was evaluated by flow cytometry for both types of cells.

**RESULTS:**

The growth rate and viability of the MSCs from the amniotic membrane was significantly higher compared with the ASCs. The low expression of CD14 and CD45 indicated that AM-MSC and ASCs are non hematopoietic cells, and both cell types expressed high percentages of CD44, CD105. The results revealed that AM-MSC and ASCs expressed no CD271 on their surfaces.

**CONCLUSION:**

This study showed that amniotic membrane is a suitable cell source for cell therapy, and CD271 is a negative marker for MSCs identification from amniotic membrane and adipose tissue.

## INTRODUCTION

Mesenchymal stem cells are derived from bone marrow,^1^ adipose tissue,^2^^,^^3^ menstrual blood,^4^ cord blood,^5^ and dental pulp.^6^^,^^7^ Multipotency and self-renewal of MSCs make them attractive for clinical application.^8^ In recent years, fetal material and amniotic membrane have attracted the attention of scientists.^9^ Amniotic membrane is a postnatal organ discarded after birth; the collection of cells does not require an invasive procedure as ethical issues are concerned.^10^ The cells derived from this tissue possess multipotent properties between embryonic and adult stem cells. In addition, immune-modulatory properties of MSCs make these cells as the primary choice for allo and xeno transplantation.^11^

Multi differentiation potential, adherence to plastic and specific surface antigen expression (CD73, CD105, CD90, and CD 44) and absence of (CD45, CD14) are three criteria defining MSCs in culture.^12^ However, researchers direct their efforts to find a suitable marker to ensure their isolation. Some studies show that CD271 (LNGFR) is one of the most selective markers for MSCs isolation from bone marrow.^13^ Cuevaz *et al.* showed that amount of CD271+ cells in ASCs correlates inversely with donor age.^14^ Iorocha *et al.* revealed that CD 271+ and CD105+ population are highly rich in molecular marker of early osteo and adipo progenitors.^15^

Quirici *et al.* published that CD 271 is a specific marker for the isolation of MSCs from bone marrow.^13^ However, Battula *et al.* reported that CD271 is not a suitable marker for MSCs isolation from chorionic membrane.^16^ Torales* et al.* proposed the use of single marker, CD271 for isolation of MSCs.^17^ The present study set to introduce a suitable source of MSCs with high proliferation rate, and to find an appropriate marker for MSCs characterization. To achieve these, MSCs properties of adult adipose tissue and amniotic membrane were evaluated.

## MATERIALS AND METHODS

Three samples of adult adipose tissue were obtained from adult patients aged 45 and 60 years old who underwent the elective surgical procedure, and samples of amniotic membrane were collected from women donors immediately after caesarean operation with informed consents and ethical approval committee of the Tabriz University of Medical Sciences. 

Under sterile condition, the samples were washed three times with phosphate buffered saline (PBS) containing 1% penicillin/streptomycin (Gibco, USA); the samples were cut into 1-2 mm thick. For enzymatic digestion, the pieces were transferred to conical tubes containing 2.5% collagenase 1 (sigma, USA) and were then shaken in water bath for 1 hour at 37°C. After digestion, for neutralizing the collagenase enzyme, the same volume of working culture medium (DMEM/Gibco, USA) containing 10% FBS (Sigma, USA) and 1% penicillin/streptomycin (Gibco, USA) were added to the cell suspension. The digested samples were centrifuged at 1600 rpm/5 min, and then cell pellet was obtained. The cells were counted under an invert microscope before plating cells in culture flasks as follows: Cells/ml=(Number of viable cells)/(Number of squares counted×dilution factor×10^4^), Total cell yield=(Cells/ml)×(Total volume of cell suspension). 

For expansion, the cells were plated at 7-10 ×10^4^ cells per T25 flask and kept at 37°C and 5% CO2. The first medium change was performed after 48 hours, and the following medium changes were three times per week. After the cells reached 80% confluence, the cells were passaged. For passaging of cells, culture medium was taken away, and then the cells were washed twice with sterile PBS (Sigma, USA). Afterwards, 1 ml of trypsin/EDTA solution was added to culture flasks, and the flasks were incubated at 37°C for 1 minute. One milliliter of working DMEM was added to the flasks for neutralizing of trypsin/EDTA. Then, the cells were centrifuged at 1600 rpm/5 min and counted for adipose and amniotic membrane cells. DT was calculated by the following formula, while N_0_ is the number of seeded cells at time t_0_, and N_t_ is the number of taken cells at time t, and T represents the culture period:

$T_{d}=T\times \frac{\log2}{\log\left(\frac{N_{t}}{N_{0}}\right)}$

At first, adipose and amniotic membrane cells were cultured in 96-well plate (3000 cells per well). After 24 hours, 300 µl pure DMEM medium and 20 µl of MTT solution (5 mg/ml, sigma USA) were added onto the cells. After four hours, 100µl of dimethyl sulfoxide (DMSO, sigma, Germany) was added onto the crystals. In a dark room, the color absorbance was recorded by ELISA reader at 540 nm.

To analyze the expression of specifics surface antigens, 10^5^ cells from each sample were washed two times with PBS containing 1% BSA and then were incubated, in appropriate combination, with 3 µl of the following fluorochrome conjugated monoclonal antibodies: CD44-FITC, CD105-PE, and CD45-FITC, CD14-PE, and CD271-PE for 30 min in a dark room. After staining, the cells were washed with PBS containing 1% BSA and centrifuged and the cell pellet vertexes with 500 µl of PBS/1% BSA were evaluated using BD instrument. 

The viability, proliferation rate during expansion period, expression of positive markers (CD44, CD105), negative markers (CD45, CD14), and expression of CD271 on MSCs surface derived from adult adipose tissue and amniotic membrane were compared. The data were analyzed using WIN MDI software. Statistical analyses were performed using Graph Pad Prism. Mean comparison was carried out via t-test analysis of variance; P<0.05 was considered significant.

## RESULTS

**Fig. 1:**
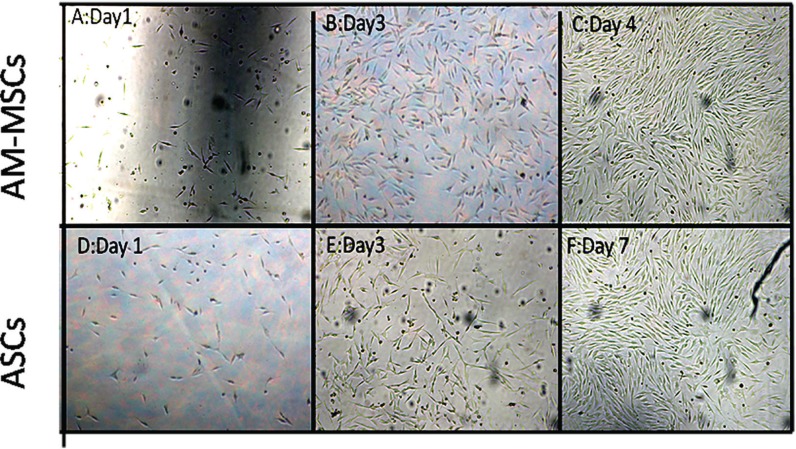
Morphology of MSCs in primary cultures, AM-MSCs at A: day 1, B: day 3, C: day4. (Magnification, ×10); AM-MSCs reached confluency at day 4. ASCs at D: Day 1, E: Day 3, F: Day 7; ASCs reached confluency at day 7

**Fig. 2:**
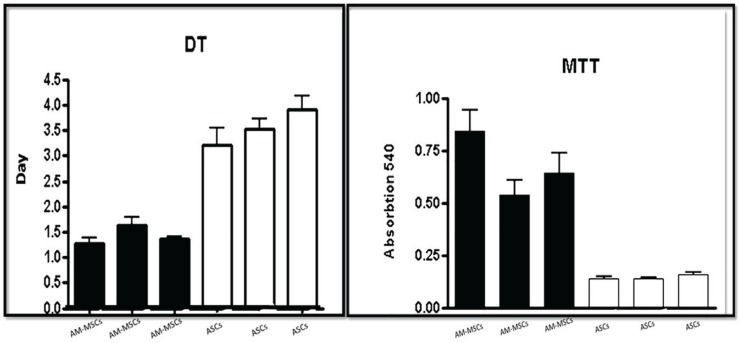
Population doubling time of ASCs compared with AM-MSCs. The growth rate of AM-MSCs was significantly higher than ASCs *(*P<0.05). MTT assays showed more active cells in AM-MSCs.

Cell expansion cultures showed that the confluence reached in the MSCs derived adult adipose tissue flasks on the seventh day, and in the AM-MSCs flasks, this was on day 4 for the same number of seeded cells (Figure 1). The DT data analysis showed that the growth rate of the AM-MSCs was significantly higher than ASCs (P<0.05) (Figure 2). 

**Fig. 3:**
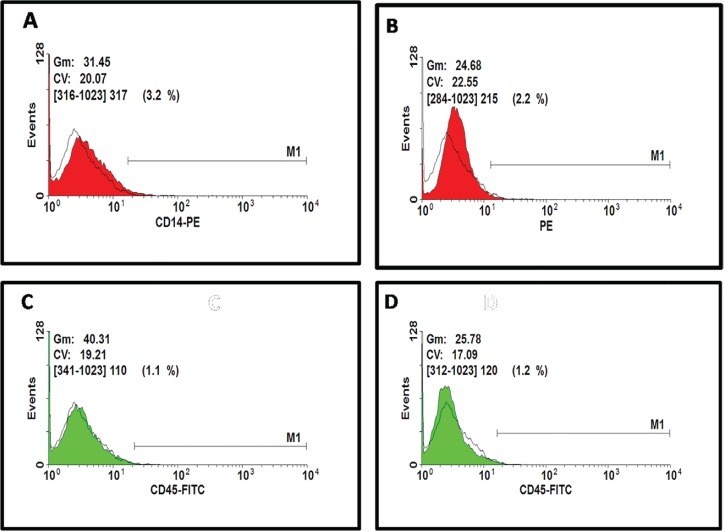
Representative results of flow cytometry analysis for negative markers: histogram depicting that (A) AM-MSCs and (B) ASCs population is negative for CD14 and histogram depicting that (C) AM-MSCs and (D) ASCs population is negative for CD45 receptor

**Fig. 4:**
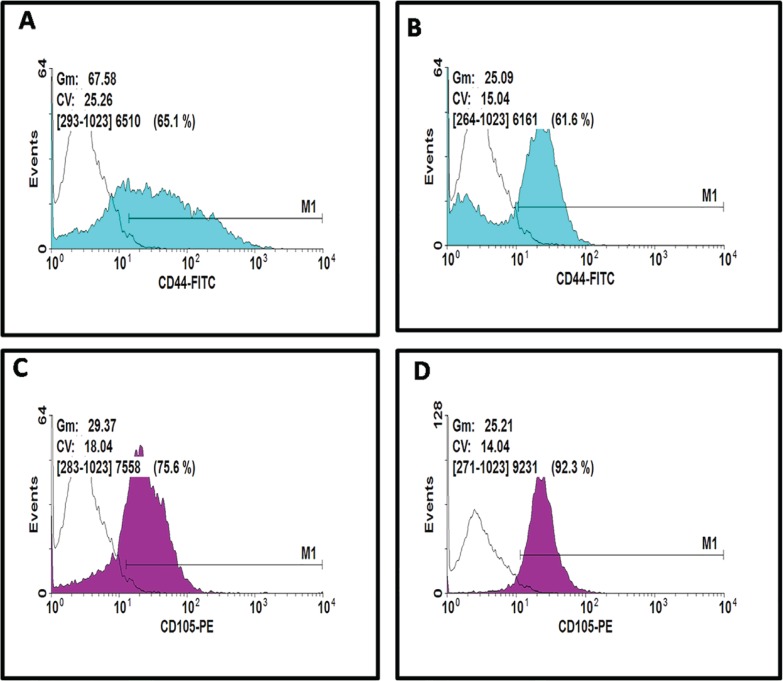
Representative results of flow cytometry analysis for positive markers**:** (A, C) histogram depicting that AM-MSCs population is positive for CD44 and CD105 receptor. (B, D) histogram depicting that ASCs population is positive for CD44 and CD105 receptor

**Fig. 5:**
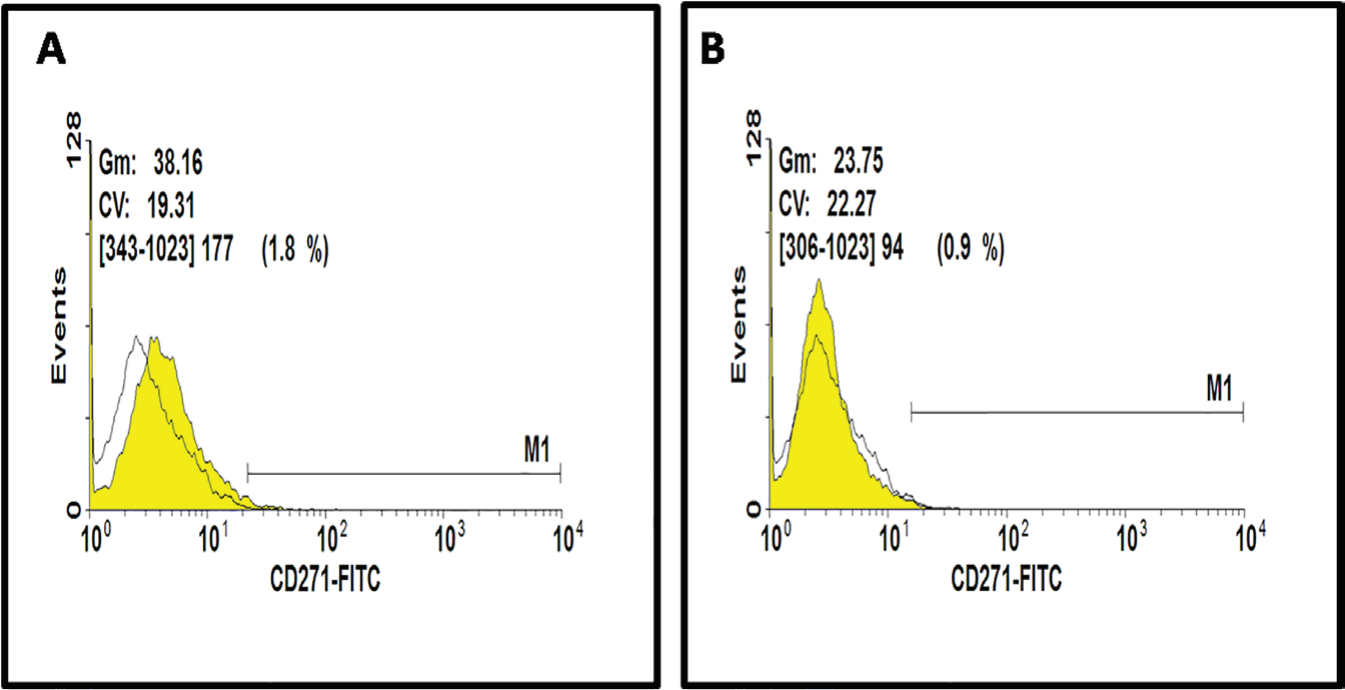
Histogram depicting that (A) AM-MSCs population and (B) ASCs population are negative for CD271 receptor

**Fig. 6:**
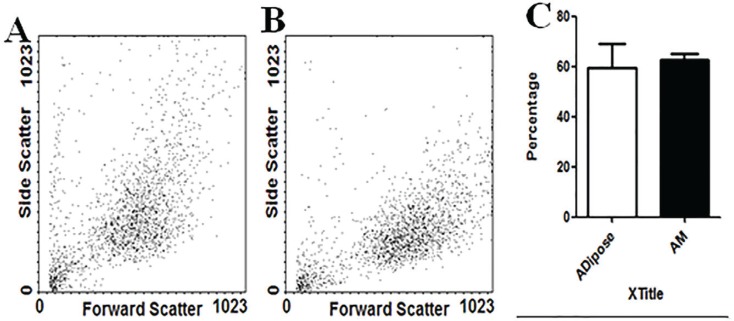
(A) AMSCs and (B) ASCs at the same in size (C) Forward scatter statistical analysis

Also, MTT assays showed more proliferation rate cells in AM-MSCs compared with ASCs; these differences were statistically significant (P<0.05) between donors (Figure 2). The analysis of expression of specific surface markers showed that AM-MSCs and ASCs population were negative regarding hematopoietic surface markers, CD14-PE and CD45-FITC (Figure 3). The histograms depict that 75.6% of AM-MSCs and 92.3% of ASCs population is positive for CD105, and 65.1% of AM-MSCs and 61/6% of ASCs population is positive for CD44 (Figure 4). Evaluation of CD271 expression showed that 1.8% of AM-MSCs and 0.9% ASCs population is positive for CD271 (Figure 5). Also the data showed that AM-MSCs and ASCs were the same in size (Figure 6).

## DISCUSSION

MSCs are the best candidates for disease treatment.^18^^,^^19^ Common limitation of MSCs in cell therapy is the low number of cells with low proliferation rate. Therefore, finding the suitable cell source is the major challenge in recent years. In this study, we concentrated on the proliferation rate and viability of MSCs from fetal material. To evaluate the efficiency of these cells in cell therapy, we compared their proliferation rate, viability, and expression of specific surface markers and CD271 expression at AM-MSCs with ASCs. The findings showed that the growth rate and viability of AM-MSCs as a fetal tissue were significantly higher than ASCs. Therefore, MSCs from fetal tissue are more suitable for therapeutic purposes compared with adult stem cells. Previous studies have not presented similar results regarding the comparison of fetal material and adult tissues.^20^

However, the current study worked on MSCs derived from bone marrow and placenta stem cells, showing no significant difference in the cell number and growth kinetics in MSCs of these sources.^21^ Hence, a new cell source was presented, overcoming the problem of low number of MSCs for cell therapy. Another limitation of the use of MSCs in clinical application is finding a suitable marker for characterization of MSCs. There are studies which show that CD271 (LNGFR) is one of the most selective markers for isolation of MSCs from bone marrow.^22^^,^^23^

In this regard, the present results revealed that ASCs and AM-MSCs express no CD271. Another study in 2013 showed that CD271^+^ ASCs correlated inversely with donor age, and the highest CD271 yield was found at 30-40 year-old range.^14^ The present inquiry proved similar results as adipose tissue samples were collected from 45 and 60 years old patients. Also, Jones *et al.* showed that CD271 is a crucial selective marker for enriching MSCs from bone marrow.^22^ Jarocha *et al.* suggested that CD271+ and CD105+ population had higher enrichment in markers of early osteo and adipo progenitors.^15^

Battula *et al. *observed that CD271 is not a suitable marker for the characterization of MSCs from chorine.^16^ Watson *et al.* published that CD271 is a suitable marker for characterization of MSCs from bone marrow, but failed to characterize MSCs from umbilical cord blood (UCB).^24^ This study presented amniotic membrane as a suitable cell source for cell therapy, also it was revealed that CD271 is a negative marker for the identification of MSCs from amniotic membrane and adipose tissue.

## References

[1] Mehrabani D, Mojtahed Jaberi F, Zakerinia M, Hadianfard MJ, Jalli R, Tanideh N, Zare S (2016). The Healing Effect of Bone Marrow-Derived Stem Cells in Knee Osteoarthritis: A Case Report. World J Plast Surg.

[2] Mehrabani D, Babazadeh M, Tanideh N, Zare S, Hoseinzadeh S, Torabinejad S, Koohi-Hosseinabadi O (2015). The Healing Effect of Adipose-Derived Mesenchymal Stem Cells in Full-thickness Femoral Articular Cartilage Defects of Rabbit. Int J Organ Transplant Med.

[3] Mehrabani D, Mehrabani G, Zare S, Manafi A (2013). Adipose-Derived Stem Cells (ADSC) and Aesthetic Surgery: A Mini Review. World J Plast Surg.

[4] Faramarzi H, Mehrabani D, Fard M, Akhavan M, Zare S, Bakhshalizadeh Sh, Manafi A, Kazemnejad S, Shirazi R (2016). The Potential of Menstrual Blood-Derived Stem Cells in Differentiation to Epidermal Lineage: A Preliminary Report. World J Plast Surg.

[5] Razmkhah F, Soleimani M, Mehrabani D, Karimi MH, Kafil-Abad SA (2015). Leukemia cell microvesicles promote survival in human umbilical cord blood hematopoietic stem cells. EXCLI J.

[6] Sholehvar F, Mehrabani D, Yaghmaei P, Vahdati A (2016). The effect of Aloe vera gel on viability of dental pulp stem cells. Dent Traumatol.

[7] Sholehvar F, Mehrabani D, Yaghmaei P, Vahdati A (2015). Survival of dental pulp stem cells: the effect of soymilk and milk. J Fasa Univ Med Sci.

[8] Aghamir SMR, Mehrabani D, Amini M, Mosleh Shirazi MA, Nematolahi S, Shekoohi-Shooli F, Mortazavi SMJ, Razeghian Jahromi I (2016). The regenerative effect of bone marrow-derived stem cells on cell count and survival in acute radiation syndrome. World J Plast Surg.

[9] S Eskandarlou M, Azimi M, Rabiee S, Seif Rabiee MA (2016). The healing effect of amniotic membrane in burn patients. World J Plast Surg.

[10] Sedighi A, Mehrabani D, Shirazi R (2016). Histopathological evaluation of the healing effects of human amniotic membrane transplantation in third-degree burn wound injuries. Comp Clin Pathol.

[11] Mortazavi SM, Shekoohi-Shooli F, Aghamir SM, Mehrabani D, Dehghanian A, Zare S, Mosleh-Shirazi MA (2016). The healing effect of bone marrow-derived stem cells in acute radiation syndrome. Pak J Med Sci.

[12] Tamadon A, Mehrabani D, Rahmanifar F, Raayat Jahromi AR, Panahi M, Zare S, Khodabandeh Z, Razeghian Jahromi I, Tanideh N, Dianatpour M, Ramzi M, Koohi-Hoseinabadi O (2015). Induction of spermatogenesis by bone marrow-derived mesenchymal stem cells in busulfan-induced azoospermia in hamster. Int J Stem Cells.

[13] Quirici N, Soligo D, Bossolasco P, Servida F, Lumini C, Deliliers GL (2002). Isolation of bone marrow mesenchymal stem cells by anti-nerve growth factor receptor antibodies. Exp Hematol Oncol.

[14] Cuevas-Diaz Duran R, González-Garza MT, Cardenas-Lopez A, Chavez-Castilla L, Cruz-Vega DE, Moreno-Cuevas JE (2013). Age-related yield of adipose-derived stem cells bearing the low-affinity nerve growth factor receptor. Stem Cells Int.

[15] Jarocha D, Lukasiewicz E, Majka M (2008). Adventage of mesenchymal stem cells (MSC) expansion directly from purified bone marrow CD105+ and CD271+ cells. Folia Histochem Cytol.

[16] Battula VL, Treml S, Abele H, Bühring HJ (2008). Prospective isolation and characterization of mesenchymal stem cells from human placenta using a frizzled‐9‐specific monoclonal antibody. Differentiation.

[17] Flores-Torales E, Orozco-Barocio A, Gonzalez-Ramella OR, Carrasco-Yalan A, Gazarian K, Cuneo-Pareto S (2010). The CD271 expression could be alone for establisher phenotypic marker in Bone Marrow derived mesenchymal stem cells. Folia Histochem Cytol.

[18] Mehrabani D, Hassanshahi Ma, Tamadon A, Zare S, Keshavarz S, Rahmanifar F, Dianatpour M, Khodabandeh Z, Jahromi I, Tanideh N, Ramzi M, Aqababa HR, Kuhi-Hoseinabadi O (2015). Adipose tissue-derived mesenchymal stem cells repair germinal cells of seminiferous tubules of busulfan-induced azoospermic rats. J Hum Reprod Sci.

[19] Álvarez-Viejo M, Menéndez-Menéndez Y, Otero-Hernández J (2015). CD271 as a marker to identify mesenchymal stem cells from diverse sources before culture. World J Stem Cells.

[20] Miao Z, Jin J, Chen L, Zhu J, Huang W, Zhao J (2006). Isolation of mesenchymal stem cells from human placenta: comparison with human bone marrow mesenchymal stem cells. Int J Biol.

[21] Jones E, McGonagle D (2008). Human bone marrow mesenchymal stem cells in vivo. Rheumatology.

[22] Kuçi Z, Kuçi S, Zircher S, Koller S, Schubert R, Bönig H (2011). Mesenchymal stromal cells derived from CD271+ bone marrow mononuclear cells exert potent allosuppressive properties. Cytotherapy.

[23] Kuçi S, Kuçi Z, Kreyenberg H, Deak E, Pütsch K, Huenecke S (2010). CD271 antigen defines a subset of multipotent stromal cells with immunosuppressive and lymphohematopoietic engraftment-promoting properties. haematologica.

[24] Watson JT, Foo T, Wu J, Moed BR, Thorpe M, Schon L (2013). CD271 as a marker for mesenchymal stem cells in bone marrow versus umbilical cord blood. Cells Tissues Organs.

